# Patient Safety Culture in Hospital Settings During the COVID-19 Pandemic: Protocol for a Scoping Review

**DOI:** 10.2196/92882

**Published:** 2026-10-22

**Authors:** Gabriele Sole, Diego Primavera, Giulia Cossu, Alessandra Perra, Orsola Marra, Massimo Tusconi, Federica Sancassiani, Monica Pedron, Paolo Albino Ferrari, Cesar Ivan Aviles Gonzales, Mauro Giovanni Carta, Antonio Macciò

**Affiliations:** 1 Department od Medical Sciences and Public Health, University of Cagliari, Cittadella Universitaria di Monserrato Strada Provinciale 8 09042 Monserrato CA, Cagliari, Sardinia, Italy, 39 070 675 3120; 2 Department of Obstetrics and Gynecology, Azienda Ospedaliera G. Brotzu, Cagliari, Sardinia, Italy; 3 PhD Program in Capacities Building for Global Health, University of Cagliari, Cagliari, Sardinia, Italy; 4 Centre for Liaison Psychiatry and Psychosomatics, Azienda Ospedaliero-Universitaria Cagliari, Cagliari, Sardinia, Italy; 5 Medical Services Management, Azienda Ospedaliero-Universitaria Cagliari, Cagliari, Sardinia, Italy; 6 Division of Thoracic Surgery, A. Businco Cancer Center, Azienda Ospedaliera G. Brotzu, Cagliari, Sardinia, Italy; 7 Università degli Studi di Enna Kore, Enna, Sicily, Italy; 8 Department of Nursing, Popular University of Cesar, Valledupar, Cesar Department, Colombia; 9 PhD Program in Tropical Medicine, Popular University of Cesar, Valledupar, Cesar Department, Colombia; 10 Department of Oncological Surgery, Azienda Ospedaliera G. Brotzu, Cagliari, Sardinia, Italy; 11 Department of Surgical Science, University of Cagliari, Cagliari, Sardinia, Italy

**Keywords:** patient safety, patient safety culture, health personnel, COVID-19, public health, disaster medicine, hospitals health care organization

## Abstract

**Background:**

Patient safety culture (PSC) is a foundational component of quality health care. It is characterized by health care workers’ individual attitudes, team dynamics, and organizational practices that influence clinical outcomes. During the COVID-19 pandemic, hospitals worldwide faced sudden changes and crises. In this rapidly evolving environment, organizational models were revised, established procedures were challenged, and professional roles were redefined; therefore, many aspects of safety culture may have been reshaped and redefined by the pandemic. Despite a growing body of literature examining PSC during the COVID-19 era, no comprehensive synthesis has yet mapped how PSC was conceptualized, measured, operationalized, and sustained across hospital settings during the pandemic. This gap highlights the need for an integrated synthesis that captures the multiple dimensions of PSC as they were experienced and managed during the pandemic.

**Objective:**

This scoping review aims to map the literature on PSC in hospital settings during the COVID-19 pandemic. The specific objectives are to examine the theoretical frameworks and related constructs used to define and operationalize PSC during the pandemic; identify and categorize interventions implemented to strengthen or sustain PSC; describe monitoring activities, measurement instruments, and quality improvement approaches adopted in hospital settings; and synthesize the scope and characteristics of available evidence and identify knowledge gaps to inform future research and practice.

**Methods:**

This scoping review will follow the methodological framework developed by Arksey and O’Malley, as updated by the JBI, and will be reported according to the PRISMA-ScR (Preferred Reporting Items for Systematic Reviews and Meta-Analyses Extension for Scoping Reviews) guidelines. The research question was formulated using the population, concept, and context framework. Searches were conducted in the PubMed, Embase, and PsycInfo databases for records published from January 1, 2020, onward. Eligible evidence sources will include peer-reviewed empirical, interventional, measurement, review, and theoretical or conceptual publications addressing PSC or safety climate in hospital care during the COVID-19 pandemic. A total of 2 independent reviewers will conduct a 2-stage screening process, initially evaluating titles and abstracts for potential relevance, followed by a full-text assessment to determine final eligibility.

**Results:**

This study commenced in April 2025 and is registered with the Open Science Framework. Database searches were completed in July 2025. Title and abstract screening commenced in July 2026. Full-text screening and data extraction are expected to be completed by September 2026, data synthesis by November 2026, and submission of the review findings in the fourth quarter of 2026.

**Conclusions:**

This scoping review will provide a structured overview of how PSC was maintained, adapted, or redefined in hospital settings during the COVID-19 pandemic. Mapping pandemic-era evidence on PSC may help hospitals translate crisis-related organizational learning into preparedness strategies, resilient safety systems, and quality improvement priorities for future public health emergencies.

## Introduction

### Overview

The World Health Organization (WHO) [1] defines patient safety as “the prevention of errors and adverse effects to patients associated with health care.” The Institute of Medicine, in its report *To Err is Human: Building a Safer Health System* [2], identified patient safety as “a critical concern within healthcare organizations and an essential first step toward improving the quality of care.” The report emphasized the importance of fostering a culture of safety within health care organizations to enhance the safety and quality of care, thereby preventing serious harm to both patients and health care professionals. Establishing such a culture has become a top priority for health care systems [3]. Patient safety culture (PSC) or safety culture refers to “the core values and behaviors resulting from a collective and sustained commitment by organizational leadership, managers, and health care workers to emphasize safety over competing goals” [4]. It is thus embedded within a conceptual framework informed by cultural theories, social dynamics, and individual behaviors in day-to-day practice [5-7]. Safety culture in health care stems from organizational culture and the collective behaviors of individuals. As such, it can be broadly described as the integrated effect of elements such as values, attitudes, perceptions, competencies, and individual and group behavioral patterns [8]. PSC constitutes a theoretical construct with practical implications, serving as a key point of reference for hospital organizational cultures [9]. While these concepts may initially be expressed at the level of the individual health care worker, they extend to all organizational levels and departments within a health care facility, ultimately influencing staff actions and behaviors.

PSC is a multifaceted construct that encompasses both individual and systemic dimensions. Operationally, it is expressed through a just culture (balancing individual accountability with a systems-based approach); open communication; psychological safety; effective teamwork; and mechanisms for reporting, analyzing, and learning from adverse events and near misses. These structural dimensions shape safety-related behaviors, influence clinical processes, and ultimately affect patient outcomes [10,11]. However, health care workers’ perceptions and behaviors remain at the core of PSC, particularly in high-pressure settings such as during the COVID-19 pandemic. Safety in health care does not rely solely on technical skills but is significantly influenced by human and organizational factors that govern everyday clinical practice [10,12]. When PSC is positive, health care workers tend to act more effectively and in a safety-oriented manner, leading to improved clinical outcomes [11,12]. Understanding how PSC was maintained, adapted, or disrupted during the COVID-19 emergency is essential for informing future preparedness strategies and supporting resilient health systems [12].

Delivering safe, high-quality care with positive outcomes must remain a central goal of health care systems even during times of crisis, such as natural disasters, conflicts, or pandemics [11]. However, the COVID-19 pandemic has significantly impacted all work environments [13], including the health care sector, introducing organizational, individual, and interorganizational challenges. COVID-19 has placed an extraordinary burden on global health care systems, profoundly affecting the provision of health care services [11].

In crisis contexts, safety culture may be disrupted, reinforced, or redefined. During the COVID era, hospital settings experienced rapid changes in care delivery models, including increased workloads, staff redeployment, the suspension of routine services, and the need to treat patients affected by a novel disease. Working under such challenging conditions may compromise health care professionals’ ability to deliver safe and effective care and has posed an unprecedented challenge for health systems worldwide [11].

Specifically, the COVID-19 pandemic has introduced multidimensional challenges, such as new requirements for continuous professional development, the implementation of processes and guidelines, and a significant increase in psychological stress among health care workers [12]. The implementation of new practices (or the modification of existing ones) within organizations, services, and systems necessitates behavioral changes at both individual and collective levels. Such a change requires a thorough understanding of the factors influencing behavior within the context in which it occurs [14].

Despite the numerous challenges posed by the COVID-19 pandemic to health care systems [15], few studies have examined changes in PSC among hospital staff during this period [16]. Nevertheless, it is essential to identify the factors that may have contributed to such changes. Attention to detail during so-called “disaster” situations is valuable for identifying common factors that make organizations vulnerable to failure [17].

### Rationale of the Study

Several studies conducted during the COVID-19 pandemic have examined specific dimensions of PSC and evaluated interventions aimed at strengthening safety-related behaviors in hospital settings [11-15]. These studies underscore the central role of core safety culture components in sustaining a positive safety culture, particularly during crises [16-18]. However, the available evidence has often been fragmented, focusing on single institutions, local initiatives, or individual-level determinants [11-15]. Moreover, although recent research has begun to describe how organizational culture and safety practices adapted during the pandemic, a unified theoretical framework capable of capturing these transformations at a broader, system-wide level still appears to be lacking [18,19]; it remains unclear whether and how existing theoretical models of PSC were adapted, challenged, or expanded during the pandemic. Given the relevance of this topic, a comprehensive synthesis of the existing evidence is warranted. This scoping review seeks to address this gap by systematically mapping how PSC has been conceptualized, assessed, or modified during the COVID-19 emergency and whether prevailing theoretical models have been revised or challenged in light of the systemic disruptions imposed by the crisis. Given the exploratory nature of this topic, the review is warranted to capture the breadth of evidence across various study designs and contexts and could identify evidence gaps relevant to future research, hospital preparedness, and quality improvement.

### Objectives

This review aims to synthesize studies that have assessed aspects of PSC in hospital settings during the COVID-19 pandemic, identifying models, determinants (individual, organizational, and interorganizational), barriers and facilitators, and specific elements and variables that constitute the construct of safety culture. Specifically, the review aims to examine (1) how safety culture was perceived and/or modified in the pandemic context; (2) which interventions, barriers, facilitators, and measurement tools were adopted by health care organizations; and (3) whether specific actions were implemented to understand how health care professionals perceived safety culture.

### Specific Objectives

The study objectives are as follows: (1) to map the theoretical models and related constructs (eg, safety climate, organizational learning, and organizational resilience) used to conceptualize PSC during the pandemic and to analyze how these models were revised, adapted, or challenged in response to organizational constraints and potential shifts in priorities within hospital settings; (2) to identify and categorize hospital-based interventions implemented during COVID-19 to strengthen or sustain PSC, including training initiatives, communication strategies, leadership actions, reporting practices, teamwork interventions, and staff support measures; (3) to describe monitoring activities, measurement instruments, and quality improvement approaches used in hospitals during the pandemic (eg, surveys and tools, audits, indicators, debriefings, huddles, and learning systems), detailing their key features, target groups, and implementation modalities; and (4) to synthesize the nature, scope, and characteristics of available evidence (study designs, settings, professional groups involved, reported outcomes, and recurring thematic areas) and to identify knowledge gaps to inform future research and evaluation or intervention priorities in crisis contexts.

## Methods

### Overview

This scoping review will be conducted following the JBI methodological guidance for scoping reviews [20] and will be reported in accordance with the PRISMA-ScR (Preferred Reporting Items for Systematic Reviews and Meta-Analyses Extension for Scoping Reviews) [21] guidelines. The original framework developed by Arksey and O’Malley [22] informed the subsequent development of the JBI approach. This scoping review protocol is registered with the Open Science Framework (OSF) [23] to ensure transparency and traceability of the process.

### Methodological Framework

The research question has been formulated according to the population, concept, and context (PCC) model recommended by the JBI.

#### Primary Research Question

The primary research question of this scoping review is as follows: What factors influenced PSC in hospital settings during the COVID-19 pandemic, and how were the theoretical frameworks used to conceptualize PSC adapted, challenged, or expanded in response to the pandemic?

#### Secondary Research Questions

The secondary research questions are as follows: (1) Which hospital-based interventions and quality improvement approaches were implemented to strengthen or sustain PSC, and what barriers, facilitators, and reported outcomes were identified? (2) Which measurement instruments and monitoring approaches were most frequently used to assess PSC in hospital settings during the COVID-19 pandemic? (3) How did pandemic-related organizational changes affect health care workers’ perceptions and practices related to PSC? and (4) What methodological limitations and knowledge gaps remain in the available evidence?

### PCC

The population includes health care professionals working in hospital settings, including physicians, nurses, allied health professionals, managers, and other hospital staff involved in care delivery, management, or safety-related processes. The concept includes PSC, including related constructs such as safety attitudes, organizational learning, just culture, psychological safety, teamwork, communication, reporting and learning from adverse events, organizational resilience, and safety-related quality improvement. The context includes hospital care during the COVID-19 pandemic or pandemic-related emergency period, regardless of the hospital unit, specialty, or the country’s income level.

### Eligibility Criteria

Eligibility criteria were defined to ensure alignment with the PCC framework while allowing inclusion of heterogeneous evidence sources, consistent with the mapping purpose of a scoping review (Table 1).

Data extracted from machine-translated reports will be independently verified by a second reviewer against the translated full text. Where translation of information relevant to eligibility, data extraction, or interpretation is unclear or ambiguous, additional linguistic verification will be sought before a final decision is made. The original language, translation method, and any additional verification procedures used for included non-English studies will be documented in the final review.

**Table 1.** Eligibility criteria according to the population, concept, and context (PCC) framework.

Domain	Inclusion criteria	Exclusion criteria
Population	Studies involving health care professionals or hospital staff involved in clinical care, management, or safety-related processes. For theoretical papers and reviews, sources will be eligible if they explicitly address health care professionals or hospital organizational safety culture in relation to patient safety.	Studies focused exclusively on patients, caregivers, students, community workers, or non–health care populations, unless data on hospital health care professionals are reported separately
Concept	Sources addressing PSC^a^, safety climate, safety attitudes, organizational safety culture, reporting and learning systems, teamwork, communication, leadership for safety, psychological safety, just culture, organizational learning, organizational resilience, or safety-related quality improvement (perceptions, barriers, facilitators, and/or interventions specifically targeting PSC). Studies using tools, methods, or approaches to evaluate, measure, or describe PSC during the COVID-19 pandemic.	Sources addressing constructs not explicitly linked to PSC or safety climate, such as general occupational stress, burnout, job satisfaction, or well-being without a clear PSC or safety climate component
Context	Studies conducted in hospital settings during the COVID-19 pandemic or pandemic-related emergency period. Studies published after May 5, 2023, will be eligible only if they report data, experiences, interventions, organizational adaptations, or conceptual reflections related to the pandemic or pandemic-related emergency period [24].	Studies conducted exclusively in primary care, long-term care, residential care, community settings, outpatient-only settings, or nonhospital settings, unless hospital data are reported separately
Evidence sources	Empirical qualitative, quantitative, and mixed methods studies; interventional and quality improvement studies; measurement studies; theoretical or conceptual papers; and reviews published in peer-reviewed journals	Conference abstracts, proceedings, editorials without analytic content, letters without original or conceptual contribution, gray literature, protocols without results or conceptual development, and non–peer-reviewed reports
Time frame	Sources published from January 1, 2020, will be considered.	Sources published before January 1, 2020
Language	No language restrictions will be applied to minimize language bias and capture evidence from diverse geographical contexts. Titles; abstracts; and, where necessary, full texts published in languages not understood by the reviewers will be translated using Google Translate and independently assessed by 2 reviewers according to the predefined eligibility criteria [25-27].	No language-based exclusion criterion will be applied.

a PSC: patient safety culture.

### Search Strategy

The search strategy was developed in accordance with the JBI guidance for scoping reviews and combined controlled vocabulary terms and free-text keywords related to PSC or safety climate. Rather than introducing additional mandatory Boolean blocks for COVID-19, hospital settings, or health care workers, the search was deliberately kept broad within a predefined publication period.

The strategy was developed for PubMed (MEDLINE) and then adapted for Embase and PsycInfo according to the controlled vocabulary and syntax of each database. The database-specific search strategies, including the search platform, controlled vocabulary, free-text terms, limits, Boolean operators, date of execution, and number of records retrieved, are provided in Multimedia Appendix 1.

The literature search was conducted in three stages, in accordance with JBI recommendations: (1) a preliminary search in the PubMed database to identify indexed terms and relevant keywords found in titles, abstracts, and article descriptors; (2) a comprehensive search in the main databases—PubMed, Embase, and PsycInfo—using a combination of controlled vocabulary (MeSH, Thesaurus, or Emtree) and free-text terms; and (3) a manual search of the reference lists of selected studies to identify additional relevant sources.

The search strategy was developed with the support of an experienced researcher and validated by a second reviewer.

A publication date limit from January 1, 2020, to July 21, 2025, was applied to delimit the predefined temporal scope of the search. July 21, 2025, represents the final search cutoff date for the present review.

The WHO declaration on May 5, 2023, that COVID-19 no longer constituted a Public Health Emergency of International Concern was used as a conceptual boundary for the emergency phase rather than as an automatic end date for publication eligibility [24]. This publication window was selected to capture both studies published during the acute pandemic phase and studies conducted during the COVID-19 emergency but published later because of the time required for data analysis, manuscript preparation, peer review, and publication. Therefore, studies published after May 5, 2023, were included only if they reported data collected during the pandemic or pandemic-related emergency period, or if they explicitly analyzed pandemic-era experiences, interventions, organizational adaptations, or conceptual developments related to PSC or safety climate in hospital settings.

### Study Selection

The search results were imported into Rayyan (Rayyan Systems Inc), a web-based tool for systematic and scoping review screening, and duplicates were removed before screening [28]. Study selection was conducted in 2 independent phases by 2 reviewers: title and abstract screening based on the predefined eligibility criteria and full-text screening of potentially relevant articles.

Before initiating the screening process, 2 research team members tested the inclusion and exclusion criteria on a random sample of 2000 retrieved records. After reaching consensus on the final criteria, titles and abstracts were screened simultaneously to identify eligible studies. Two authors (GS and OM) independently applied the inclusion criteria to all retrieved citations. Articles selected by both reviewers will proceed to full-text review for the final inclusion decision. Any discrepancies were resolved through discussion with a third reviewer (AP).

### Quality Appraisal

Consistent with the exploratory and mapping purpose of scoping reviews, methodological quality appraisal will be conducted for descriptive and interpretive purposes only. Quality appraisal findings will not be used to determine study inclusion or exclusion. All sources that meet the PCC-based eligibility criteria will be included in the review regardless of their methodological quality appraisal results.

The rationale for conducting a descriptive quality appraisal is to provide additional context on the methodological characteristics, strengths, and limitations of the available evidence. This is particularly relevant because the review is expected to include heterogeneous evidence sources, including observational, interventional, qualitative, and mixed methods studies conducted in hospital settings during the COVID-19 pandemic.

Quality appraisal will not affect eligibility, weighting, or inclusion in the synthesis. Primary empirical studies will be appraised using the design-appropriate National Institutes of Health tools or the Critical Appraisal Skills Programme qualitative checklist [29,30]. For mixed methods studies, each component will be appraised using the corresponding tool. Reviews and conceptual papers will be synthesized separately and will not undergo these design-specific appraisals. Two reviewers will independently conduct the appraisals, with disagreements resolved through consensus or consultation with a third reviewer.

Two authors will independently assess the quality of included studies. Any discrepancies will be resolved through discussion and consensus with a third author.

### Data Extraction

Data from included sources will be extracted using a standardized data extraction form developed by the review team and adapted from the JBI template for scoping reviews. The form will be designed to ensure explicit alignment between the review objectives, extracted data fields, and planned synthesis. Before full data extraction, the form will be pilot-tested using a sample of included sources and refined through discussion among the review team to improve clarity, consistency, and completeness.

Two reviewers will independently chart data using a JBI-informed form explicitly aligned with the review objectives (Table 2). The form will be pilot-tested and refined before full extraction. Disagreements will be resolved by consensus or consultation with a third reviewer, and operational definitions will be documented in the final charting form.

Data will be extracted using a standardized form explicitly aligned with the review objectives (Table 2). The form will capture study characteristics, conceptual frameworks, measurement approaches, interventions, implementation factors, and evidence gaps.

The extraction form will be pilot-tested using a sample of included sources and refined by the review team before full data extraction. Detailed operational definitions and coding instructions will be documented in the final data charting form.

**Table 2.** Data extraction and synthesis framework by review objective.

Review objective or cross-cutting analytical domain	Main data extraction fields	Purpose in synthesis
Objective 1: theoretical models	Definition of PSC^a^ or safety climate, theoretical framework and related constructs, level of analysis, and COVID-19–related adaptations or conceptual developments	To compare how PSC was defined, operationalized, adapted, or reframed during the pandemic
Objective 2: interventions	Intervention or quality improvement type, target group and setting, PSC domain, implementation strategy and timing, outcomes, and barriers and facilitators	To classify interventions according to type, target, implementation features, PSC domain, and reported outcomes
Objective 3: measurement and monitoring	Instrument and domains assessed; population, administration mode, and timing; monitoring, audit, feedback, reporting, or learning activities; and pandemic-related adaptations	To map how PSC was measured, monitored, and incorporated into quality improvement processes
Objective 4: evidence characteristics and gaps	Authors, year, and country; study design and source type; hospital setting and sample; pandemic phase; and main findings, limitations, implications, and evidence gaps	To describe the distribution and characteristics of the evidence and identify methodological and knowledge gaps
Cross-cutting factors	Individual-, team-, organizational-, and system-level determinants and pandemic-related effects on workload, workflows, communication, teamwork, leadership, reporting, staff support, and safety priorities	To identify recurring barriers, facilitators, and determinants of PSC across the review objectives

a PSC: patient safety culture.

### Data Analysis and Synthesis

A structured synthesis framework will be applied to organize findings according to the review objectives. Theoretical and conceptual contributions will be synthesized by identifying the definitions, models, and related constructs used to describe PSC during the pandemic, including organizational learning, just culture, psychological safety, teamwork, communication, and organizational resilience. Measurement tools will be compared based on instrument name, domains assessed, target population, administration modality, timing of data collection, and whether pandemic-related adaptations were reported.

Interventions and quality improvement approaches will be classified according to intervention type, target group, implementation strategy, safety culture component addressed, and reported outcomes. Qualitative and mixed methods findings will be synthesized using thematic and content analysis to identify recurring concepts, patterns, and contextual factors. Reviews and theoretical papers will be analyzed separately from primary empirical studies and used to identify existing syntheses, conceptual developments, and evidence gaps.

An initial deductive framework based on the objectives and individual-, team-, organizational-, and system-level factors will be expanded inductively when additional recurring concepts emerge. Where appropriate, results will be presented through summary tables, evidence maps, conceptual maps, and thematic matrices. Word clouds and similarity analyses will be used only as supplementary exploratory visualizations and will not replace the structured synthesis framework.

Findings will be presented using summary tables, conceptual maps, graphical representations (eg, word clouds, similarity analysis, or descending hierarchical classification), and thematic maps. The review will present the prevailing theoretical frameworks, intervention strategies, monitoring and measurement approaches, and reported outcomes organized according to the 4 specific objectives outlined in Table 2.

### Ethical Considerations

No ethics approval is required for this study, as it is based solely on data from previously published sources.

## Results

This study commenced in April 2025 and is registered with the OSF. Database searches were completed in July 2025. The searches identified 25,191 records before deduplication and 20,044 records after duplicate removal. Title and abstract screening commenced in July 2026. Full-text screening and data extraction are expected to be completed by September 2026, data synthesis by November 2026, and submission of the review findings in the fourth quarter of 2026.

## Discussion

### Principal Anticipated Findings

This scoping review is expected to provide a comprehensive map of how PSC and safety climate were conceptualized, measured, monitored, and supported in hospital settings during the COVID-19 pandemic. The review is anticipated to identify the main theoretical and conceptual models used to frame PSC in a crisis context; the most frequently adopted measurement tools; the types of interventions and quality improvement approaches implemented; and the individual-, team-, organizational-, and system-level barriers and facilitators reported in the literature.

The review may also show whether pandemic-related organizational disruptions, such as staff redeployment, increased workload, changes in care pathways, infection prevention requirements, and psychological pressure on health care professionals, were associated with changes in how PSC was understood and operationalized in hospitals. By mapping these elements, the review will clarify whether the pandemic literature mainly described safety culture through established instruments and frameworks or whether it introduced adaptations, emerging constructs, or crisis-specific perspectives.

Previous literature has described PSC as a multidimensional construct involving leadership, communication, teamwork, organizational learning, reporting practices, just culture, psychological safety, and shared safety-related values and behaviors [8-11]. During the COVID-19 pandemic, these dimensions were challenged by rapidly changing clinical demands, uncertainty, resource constraints, and changes in professional roles and organizational priorities [11,13,17,18]. Although individual studies have examined specific aspects of PSC during the pandemic, the evidence remains fragmented across countries, hospital settings, professional groups, study designs, and measurement approaches. This scoping review will address this gap by systematically mapping how available studies have defined, assessed, and interpreted PSC in hospital care during the pandemic period.

### Strengths and Limitations

The broad scope of this review may enable an integrated understanding of conceptual, measurement, and organizational aspects of PSC across diverse hospital contexts. However, the expected heterogeneity of study designs and settings may limit direct comparability, while the exclusion of gray literature may underrepresent locally implemented safety initiatives.

The literature search was closed on July 21, 2025. Consequently, studies indexed or published after this date will not be captured in the present review, including potentially relevant studies reporting pandemic-era data that were published later. This temporal cutoff should therefore be considered when interpreting the comprehensiveness of the evidence map.

### Implications and Dissemination

The findings of this review are expected to inform future research on PSC in crisis and postcrisis hospital contexts. By identifying recurring constructs, measurement tools, interventions, and evidence gaps, the review may support the development of more targeted studies, longitudinal research, and intervention evaluations. The results may also inform hospital preparedness strategies, quality improvement programs, and organizational learning processes aimed at maintaining patient safety during future public health emergencies.

The findings will be disseminated through publication in a peer-reviewed journal and presentations at scientific conferences.

### Conclusions

This scoping review will provide a structured overview of how PSC was conceptualized, measured, maintained, adapted, supported, and redefined in hospital settings during the COVID-19 pandemic. By mapping theoretical models, measurement approaches, interventions, barriers, facilitators, and evidence gaps, the findings will contribute to a better understanding of organizational resilience and learning processes during health care system crises and inform the development of evidence-based preparedness strategies and policy recommendations aimed at sustaining patient safety during future emergencies.

## Supplementary material

10.2196/92882 Multimedia Appendix 1 Complete search strategies for PubMed (MEDLINE), Embase, and PsycInfo databases.

## Abbreviations

OSF: Open Science Framework
PCC: population, concept, and context
PRISMA-ScR: Preferred Reporting Items for Systematic Reviews and Meta-Analyses Extension for Scoping Reviews
PSC: patient safety culture
WHO: World Health Organization
