# Supplementary material for: Patient Safety Culture in Hospital Settings During the COVID-19 Pandemic: Protocol for a Scoping Review
Source: JMIR Res Protoc. 2026 Oct 22;15:e92882. doi: 10.2196/92882 (PMC13717335; doi:10.2196/92882)
Supplement: Supplementary file 1 [file resprot-v15-e92882-s001.docx]

Appendix 1. Complete Search Strategies for PubMed/MEDLINE, Embase, and PsycINFO:

| Database/  Platform | B1 – Search Strategy | B2 – Publication date limit | Search execution date | Records retrived |
| --- | --- | --- | --- | --- |
| PubMed/  MEDLINE  via PubMed | ("safety management"[MeSH Terms] OR "Organizational Culture"[Title/Abstract] OR  "organizational culture"[Title/Abstract] OR  "organizational health"[Title/Abstract] OR "safety culture"[Title/Abstract] OR  "culture safety"[Title/Abstract] OR "no-blame"[Title/Abstract] OR  "organizational safety"[Title/Abstract] OR "safety competence"[Title/Abstract] OR "psychological safety"[Title/Abstract] OR  "just culture"[Title/Abstract] OR "reporting culture"[Title/Abstract] OR  "blame culture"[Title/Abstract] OR "safety values"[Title/Abstract] OR  "safety behavior"[Title/Abstract] OR  "learning culture"[Title/Abstract] OR  "hospital organization"[Title/Abstract] OR  "organizational climate"[Title/Abstract]) | 01/01/2020  To  21/07/2025 | 21/07/2025 | 6099 |
| Embase  Via Embase.com | ('safety culture'/exp OR 'safety culture':ti OR 'safety culture':ab OR  'organizational culture'/exp OR 'organizational culture':ti OR 'organizational culture':ab OR  'no-blame':ti OR 'no-blame':ab OR  'organizational safety':ti OR 'organizational safety':ab OR  'safety competence':ti OR 'safety competence':ab OR  'psychological safety':ti OR 'psychological safety':ab OR  'just culture':ti OR 'just culture':ab OR  'reporting culture':ti OR 'reporting culture':ab OR  'blame culture':ti OR 'blame culture':ab OR  'safety values':ti OR 'safety values':ab OR  'safety behavior':ti OR 'safety behavior':ab OR  'learning culture':ti OR 'learning culture':ab OR  'hospital organization'/exp OR 'hospital organization':ti OR 'hospital organization':ab OR  'organizational climate'/exp OR 'organizational climate':ti OR 'organizational climate':ab) | 01/01/2020  To  21/07/2025 | 21/07/2025 | 13615 |
| PsycINFO via EBSCOhost | (DE "Patient Safety" OR  DE "Organizational Culture" OR  TI("safety culture") OR AB("safety culture") OR  TI("organizational culture") OR AB("organizational culture") OR  TI("organizational health") OR AB("organizational health") OR  TI("no-blame") OR AB("no-blame") OR  TI("organizational safety") OR AB("organizational safety") OR  TI("safety competence") OR AB("safety competence") OR  TI("psychological safety") OR AB("psychological safety") OR  TI("just culture") OR AB("just culture") OR  TI("reporting culture") OR AB("reporting culture") OR  TI("blame culture") OR AB("blame culture") OR  TI("safety values") OR AB("safety values") OR  TI("safety behavior") OR AB("safety behavior") OR  TI("learning culture") OR AB("learning culture") OR  TI("hospital organization") OR AB("hospital organization") OR  TI("organizational climate") OR AB("organizational climate")) | 01/01/2020  To  21/07/2025 | 21/07/2025 | 5477 |
|  | total |  |  | 25191 |

The search strategy consisted of a broad conceptual block addressing patient safety culture, safety climate, and related organizational safety constructs, combined with a predefined publication date limit. COVID-19, hospital setting, and health care worker terms were not applied as mandatory Boolean blocks; their relevance was assessed during study selection according to the predefined PCC eligibility criteria.
